# Actively Controlled Exoskeletons Show Improved Function and Neuroplasticity Compared to Passive Control: A Systematic Review

**DOI:** 10.1177/21925682251343529

**Published:** 2025-06-05

**Authors:** Ka Ioi Argus Chiu, Charles Taylor, Priyanshu Saha, James Geddes, Timothy Bishop, Jason Bernard, Darren Lui

**Affiliations:** 1 4915 St. Georges University of London, UK; 2 Department of Spinal Surgery, 156611 St. Georges Hospital, NHS Foundation Trust, London, UK

**Keywords:** exoskeleton, spinal cord injury, rehabilitation, mobility, continence, pain, quality of life

## Abstract

**Study Design:**

Systematic Review.

**Objectives:**

To determine whether actively controlled exoskeletons or passively controlled exoskeletons are better at rehabilitating patients with SCIs.

**Methods:**

A literature search between January 2011 to June 2023 on Pubmed Central, Pubmed, Web of Science and Embase was carried out. Exoskeletons were classified as actively controlled if they detect bioelectrical signals (HAL). All other exoskeletons were classified as passively controlled (ReWalk, Ekso, H-MEX, Atlante, Indego, Rex Bionics, SuitX Phoenix, Lokomat and HANK). Functional outcomes used were 6 minute walk test (6MWT) distance and 10 metre walk test (10MWT) speed. Further subgroup analysis was carried out for acute and chronic SCI patients. All outcomes were examined without the aid of the exoskeleton device. Secondary outcomes including continence, pain and quality of life were also examined.

**Results:**

555 articles were identified in the initial search and 27 were included in the review resulting in a total of 591 patients and 10 different exoskeleton models. HAL was the only exoskeleton to show improvements in both mobility and all secondary health outcomes. HANK and Ekso also showed improvements in mobility. Rewalk showed improvements in all secondary health outcomes with Ekso only showing improvements in QoL. No other exoskeletons showed significant improvements.

**Conclusion:**

In conclusion, the actively controlled exoskeleton HAL showed improvement in all outcomes of interest suggesting that neuroplasticity could be induced with HAL rehabilitation allowing the weakened bioelectrical signals to transcend the SCI to show genuine improvements.

## Introduction

In the United Kingdom, it is estimated that there are 2500 cases of life changing spinal cord injuries (SCI) each year with a total of 50 000 people living with an SCI at any one time.^1^ In summary, SCI is a complex neurological condition characterised by disruption of sensory, motor, and autonomic pathways. They are broadly classified into complete injuries, where there is total loss of function below the level of injury, and incomplete injuries, where some motor or sensory function is preserved.^2^ Anatomically, injuries may result in tetraplegia (cervical level, affecting all four limbs and trunk) or paraplegia (thoracic, lumbar, or sacral level, affecting lower limbs and potentially trunk function). The aetiology of SCI can be traumatic or non-traumatic, resulting from conditions such as tumors, infections, or degenerative diseases.^3^ Pathophysiologically, SCI triggers a cascade of events including primary mechanical damage, followed by secondary injury mechanisms such as ischaemia, inflammation, excitotoxicity, and demyelination. These changes lead to neural circuit disconnection and motor impairments, often rendering patients unable to perform voluntary movements.^2^ This complex clinical and neurobiological landscape underscores the urgent need for interventions like exoskeleton-assisted rehabilitation, which can promote task-specific movement, reduce complications of immobility, and stimulate activity-dependent neuroplasticity critical for recovery.^4^

Exoskeletons are a management strategy that enables individuals with an SCI to safely and effectively mobilise and rehabilitate. Generally, exoskeletons typically use actuators (motors or pneumatics) and sensors (force, position, EMG) to detect user intent or assist movement. These devices stabilise the body and guide it through repetitive, task-specific motions, such as walking or reaching, with precise control over joint angle, torque, and timing. Biomechanically, exoskeletons reduce the load on weak muscles, enforce correct kinematic patterns, and normalise gait cycles or limb trajectories, allowing patients to practice functional movements even when they lack the strength or coordination to perform them independently. This repetitive and task-specific movement is key to facilitating neuroplasticity—the brain’s ability to reorganize and form new neural connections. By providing high-intensity, consistent sensory-motor feedback, exoskeletons enhance motor learning, cortical reorganization, and synaptic strengthening in damaged neural circuits (e.g., after stroke or SCI). Over time, this supports restoration of voluntary motor control, particularly when combined with active patient engagement and feedback-driven training.^4^

Exoskeletons can be classified based on their mechanism and how they are controlled. Strictly continuous ‘passive’ motion exoskeletons disregard the intent of the user and focus on repetitive movements without user feedback. Other exoskeletons can be controlled by bio-electrical signals such as surface electromyography which act as sensors on the skin to detect electrical activity in the intended muscle group.^5^ Exoskeletons can then detect any ‘active’ signals in the sensors to initiate and control the intended voluntary movement.^6^

Continuous movements and exercise can reactivate the central pattern generator and recircuit neurons in the spinal cord. Functional magnetic resonance imaging studies shows that active movements induces a greater neural response in the central nervous system than passive movements.^4^ Due to the nature of actively controlled exoskeletons, the robot can amplify the weakened bio-electrical signals through sensors on the patient’s skin and send these signals to the target muscle groups to produce the intended movement. This successful movement is the feedback to the central nervous system to enable it to learn and develop these signals and increase their strength.^6^ The neural circuits can adapt and regenerate to facilitate partial innervation to spinal cord segments below the level of injury making the bypass a significant factor for rehabilitation and recovery.^4^ This subsequentially improves the patient’s physical function without the assistance of the robot.^6^ On the other hand, passive controlled exoskeletons do not detect bio-electrical signals in the patient’s body. They improve the patient’s function through other mechanisms such as recognizing patterns and repetition.^7^ These exoskeletons can detect shifts in the users’ weight to initiate continuous limb movement^8^ or they can initiate movement through a handheld tablet.^9^ This passive movement is thought to induce less neural regeneration and therefore less improvements in function.^4^

To date, there are no systematic reviews comparing which exoskeletons have the greatest rehabilitative effect on mobility and secondary health outcomes in patients that have suffered an SCI. The aim of this systematic review is to determine whether active or passive exoskeleton rehabilitation is more effective with regards to patient mobility and secondary health outcomes following an SCI.

## Methods

### Data Collection

This systematic review was carried as per the Preferred Reporting Items for Systematic Reviews and Meta-analyses (PRISMA) 2020 guidelines.^10^ A literature search was conducted on 3^rd^ of July 2023 for the 1^st^ of January 2011 to 30^th^ of June 2023 on Pubmed Central, Pubmed, Web of Science and Embase. Key terms used for the search were “exoskeletons”, “spinal cord injury”, “spinal rehabilitation” and “lower limb” were combined with the Boolean Operator “AND”. In addition, the Boolean Operator “NOT” was included for “stroke”. We included all human studies excluding other systematic reviews with or without meta-analyses. All of the key terms were used for the complete search. All abstracts and full texts articles were considered if available in English. Full inclusion and exclusion criteria are shown in Table 1. All types of SCI, traumatic and atraumatic, were included in this study.

Abbreviations were not included in the search strategy as a prior literature search indicated that all full search terms were included in all manuscripts before their abbreviated use. A prior scoping search did not return a greater number of articles when including truncated or abbreviated terms.

**Figure 1.**
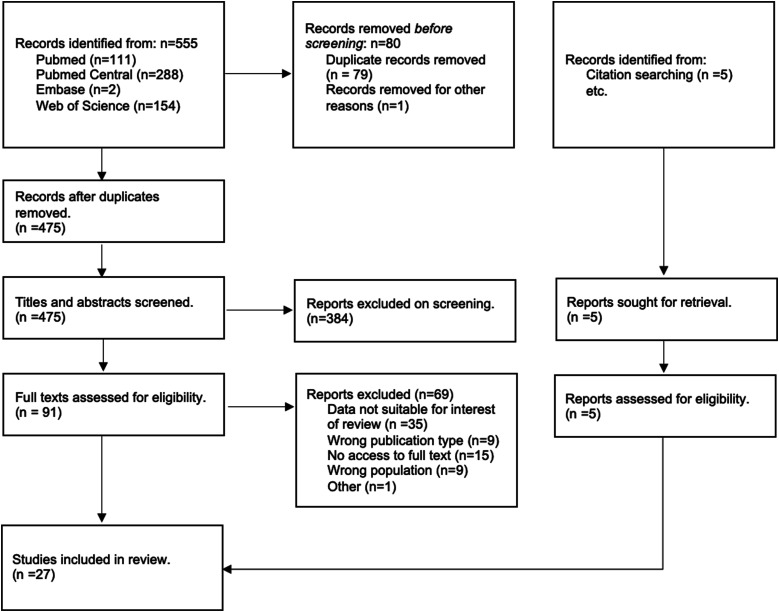
PRISMA flowchart for the review.

Duplicate records were removed using the Rayyan software.^11^ All studies were assessed for eligibility by 2 authors independently (KIAC and CT). In situations where disagreement arises, a third author (DL) is consulted. The title and abstracts were first evaluated followed by examination of the full text (Figure 1). The same author then extracted the data of interest from the studies.

### Outcomes

Based on their mechanism of action, exoskeletons were classified into passive or active rehabilitation for comparisons (Table 2). Individual study data was extracted and tabulated in Table 15 by the same authors independently.

Primary outcomes included the difference between the active and passive exoskeletons percentage change in 6 minute walking test and 10 metre walking test (6MWT and 10MWT). These were calculated from the ambulatory distance in meters for the 6MWT and speed in meters per second (m/s) for the 10MWT. To allow for comparison, all other functional mobility measures were excluded. Subgroup analysis was performed for acute vs chronic SCI patients. Secondary outcomes that were also considered included effect on continence, pain and quality of life (QoL).

Data extraction and statistical analysis was performed on SPSS (version 26.0.0.0) and Stata (version 17.0.0.0). All outcomes included descriptive statistics. When appropriate, further analytical and comparative statistics were performed. Level of evidence was graded per the Centre for Evidence-Based Medicine’s (CEBM) recommendations (Table 15, Appendix A). The quality of the studies was assessed by the same authors independently using the Newcastle-Ottawa Scale^12^ for non-ramdomised studies and the Rob2^13^ for randomised studies (Table 16 and 17, Appendix A).

### Results

A total of 555 articles were identified after the initial search. Pubmed (n = 111), Pubmed Central (n = 288), Embase (n = 2), Web of Science (n = 154), 5 additional articles were identified from reference searching. Among these, 79 articles were removed as duplicates and 1 for other reasons (Figure 1).

The title and abstracts were screened for the remaining 475 articles resulting in 384 papers excluded for not meeting the inclusion and exclusion criteria. 91 articles were retrieved for the full text review and 69 were excluded resulting in 27 articles eligible for final inclusion.

The 27 studies included a total of 591 participants, 231 motor complete injuries (ASIA A and ASIA B) and 321 motor incomplete injuries (ASIA C and ASIA D). Two studies did not specify the ASIA level for the population of interest.^14,15^ 171 patients were in the acute phase of injury (less than 1 year since injury) and 282 patients were in the chronic phase (more than 1 year since injury). Eight studies did not specify or had both acute and chronic patients which were not able to be separated for the outcome measures of interest.^8,9,15-20^

Ten exoskeleton models were identified in this review covering the 2 different types, active and passive (see Table 2 for classification). 314 patients were enrolled for rehabilitation with Hybrid Assisted Limb (HAL), 56 patients were enrolled with Lokomat, 57 patients were enrolled for Ekso, 45 patients were enrolled for ReWalk, 45 patients were enrolled for Indego, 11 patients was enrolled for HANK, 40 patients were enrolled for SuitX Phoenix, 10 patients were enrolled for H-Mex, 11 patients were enrolled for Atalante, 2 patients were enrolled for Rex Bionics.

## Primary Outcomes

### 6MWT

Eight studies including 230 patients looked at the 6MWT without the assistance of the exoskeleton post rehabilitation^21-28^ Significant improvements were seen in all except for one study^26^ (Tables 3 and 4).

### 10MWT

Thirteen studies including 329 patients looked at the 10MWT without the assistance of the exoskeleton post rehabilitation^15,18,21-31^ Significant improvements were seen in all except for 5 studies^15,23,26,29,30^ (Tables 5 and 6).

Owing to the data’s non-parametric distribution and unequal variances, a Mann-Whitney-U test was performed to compare active powered Hybrid Assisted Limbs with all passive powered exoskeletons. This analysis was performed for the combined 6MWT and 10MWT by comparing average pre- vs post-operative percentage change. The results indicate a significant difference between groups, [U = 20.00, *P* = 0.023, z = 2.281].

Individually, the 6MWT HAL group achieved on average a 39.22% greater percentage improvement (73.82%, SD:24.02 vs 34.60%, SD: 25.21), the 10MWT HAL group achieved a 26.28% greater percentage improvement (113.96%, SD:71.87 vs 87.68%, SD: 118.97). However, neither were individually statistical significance (*P* = 0.0702, df6, t = 2.1992 and *P* = 0.6256, df11, t = 0.5020).

### Subgroup Acute Vs Chronic SCI Comparison

Subgroup analysis comparing acute and chronic SCIs was also performed. For acute SCI, 3 studies including 108 patients looked at the 6MWT^21,24,27^ (Tables 7 and 8) and 5 studies including 124 patients for the 10MWT^21,24,27,31,32^ (Table 9 and 10). For the acute SCI’s, significant improvements were seen in all studies except 1 HAL study in the 10MWT which didn’t carry out a statistical test.^31^

With regards to chronic SCIs, 5 studies looked at the 6MWT^22,23,26-28^ and 6 studies for the 10MWT^22,23,26-28,32^ (Table 11–14).

## Secondary Outcomes

### Continence

Seven studies including 136 patients looked at continence (HAL n = 1, ReWalk n = 2, H-MEX n = 1, Atlante n = 1, Indego n = 1, Ekso n = 1 and Lokomat n = 1).^17,19,33-37^ 1 HAL study and 2 ReWalk studies showed statistically significant improvement in continence post rehabilitation with the exoskeleton. Brinkemper et al showed a reduction in Wexner Score from 8.89 to 6.51 with HAL (*P* = 0.008).^34^ Van nes et al showed significant improvements in bladder management using the Neurogenic Bladder Symptoms Score from 3 to 4 (*P* = 0.01).^19^ Chun et al showed significant improvements in 7 patients (defined by >10% in decrease) using the SCI-QoL bowel management difficulties short form instrument with the ReWalk. However, there was 1 patient who showed significant worsening.^35^

### Pain

Eight studies including 134 patients looked at pain (HAL n = 2, ReWalk n = 2, Indego n = 1, Ekso n = 1, Atlante n = 1, SuitX Phoenix n = 1).^8,14,17,26,29,33,38,39^ Only 1 HAL and 1 ReWalk study showed significant reductions in pain post rehabilitation with the exoskeleton. Cruciger et al showed a significant reduction in pain from 4.3 to 0.6 on the NRS scale for 2 patients and Khan et al showed a significant reduction in pain using the NRS scale for 1 patient.^14,29^

### Quality of Life

Eight studies including 125 patients looked at QoL (HAL n = 2, ReWalk n = 2, Indego n = 1, Ekso n = 1, H-MEX n = 1, Rex Bionics n = 1).^9,16,19,26,29,36,39^ One HAL, Rewalk and Ekso study showed significant improvements in QoL. 2 HAL patients improved in all domains of the Short Form 36 (SF36) and 21 ReWalk patients improved in 4 out of 8 domains in the SF36.^19,29^ 21 Ekso patients improved in the Short Form 12 (SF12) from 21.1 to 35 for ASIA A patients and 26.9 to 35.7 for ASIA B patients.^16^

## Discussion

### Primary Outcomes: 6MWT and 10MWT

Patients who have suffered an SCI often struggle with their mobility, consequentially, they are treated in a multidisciplinary approach with goals set based on patients’ injury, abilities and preferences.^40^

This review finds that the HAL, HANK and Ekso models were able to demonstrate significant improvements in the 6MWT along with HAL and HANK also showing significant improvements in the 10MWT. No other exoskeletons showed significant improvements in mobility.

On average, the active exoskeleton HAL, showed improvements of 77.8% in the 6MWT and 90.5% in the 10MWT. The passive exoskeletons on average showed improvements of 47.1% for the 6MWT and 67.6% in the 10MWT. Statistical analysis comparing the 6MWT and 10MWT for active against passive exoskeletons showed significant improvements in the active exoskeletons with a *P*-value of 0.023. This suggests that use of these active models may provide an enhanced rehabilitation pathway with a subsequent increase in their baseline function compared to passive models.

Some passive exoskeletons were able to show significant improvements in the 6MWT and 10MWT. Kim et al and Sale et al showed improvements of 138% and 103% in the 6MWT compared to baseline with the H-MEX and Ekso respectively.^8,41^ This is of a similar magnitude to the improvement in Aach et al’s HAL study of 133%.^28^ This suggests that the passive exoskeletons were also able to increase the patients’ ability to walk but only with the exoskeleton suit on. This difference could be partially attributed to the exoskeleton mechanism. For instance, the HAL uses voluntary control mechanisms, detecting bio-electrical signals from the skin surface to activate actuators, thereby enabling users to initiate movement through residual motor intent.^29^ This promotes active participation, reinforcing neuroplasticity via sensorimotor integration.^4^ In contrast, the H-MEX operates via pre-programmed gait cycles triggered by joystick or tablet interface, offering less neural engagement and relying more on passive movement assistance.^41^ HAL’s real-time feedback and adaptive control may lead to greater improvements in voluntary motor function and cortical reorganization, particularly in users with incomplete SCI. Meanwhile, H-MEX may be more suitable for patients with complete injuries, emphasizing functional mobility and safety rather than neuro-recovery. These mechanistic distinctions highlight the importance of matching device capabilities to patient profiles, which may help improve therapeutic outcomes and inform future comparative research. However, this has not been individually studied or assessed. No studies provided direct comparison at both 6MWT and 10MWT tested with and without the exoskeleton.

The 10MWT is a test of walking speed whereas the 6MWT is a test of endurance.^42,43^ With HAL, Ekso and HANK being the only exoskeletons showing significant improvements in mobility, HAL and HANK both showed a larger magnitude increase in the 10MWT from baseline compared to the 6MWT. HAL and HANK showed an average of 91% and 68% increase in 10MWT but only a 78% and 60% increase in the 6MWT respectively. The 4 patients using Ekso in Chang et al’s study did not produce significant changes in the 10MWT. This suggests that exoskeleton rehabilitation potentially improves the patients’ walking speeds in short distances. When improvements in endurance is required, the genuine improvements aren’t as significant.

In a systematic review by Tamburella et al, they have found that rehabilitation with exoskeleton devices including Ekso, Rewalk, Indego and HAL showed improvements in the 10MWT. However, they did not specify whether the improvements were carried out with or without the exoskeleton device and no comparisons were made between the active and passive exoskeleton devices.^44^

Despite not achieving statistical significance in the individual analysis, this likely represents a type II error owing to the underpowered sample and small study number. It is therefore important for future studies to further explore this relationship as a 40% improved 6MWT and 26% improved 10MWT as demonstrated in this study may be clinically significant.

### Subgroup Analysis

For both acute SCI and chronic SCI, active exoskeleton HAL showed greater improvements in the 6MWT and 10MWT compared to passive exoskeletons. HAL was also the only exoskeleton to show improvements in all 4 disciplines.

For acute SCI, active exoskeleton HAL showed greater improvements in both the 6MWT and 10MWT compared to passive exoskeletons. HAL showed improvements of 82.5% in the 6MWT compared to HANK’s 60.1%. HAL also showed improvements of 186.2% in the 10MWT compared to 67.6% and 23.7% for HANK and Ekso respectively. This suggests that active exoskeletons like HAL may be better than passive exoskeletons to improve patients walking speeds and endurance in the acute phase of rehabilitation. In addition, during the acute phase of an SCI, inflammation levels are high. Immune cells are attracted to the site of injury causing neuroinflammation and destruction of tissue.^3^ Although there are no studies that determine the optimal timing of rehabilitation,^45^ the pro-inflammatory environment may not provide the optimal conditions for neuro-regeneration. This can suggest that active exoskeletons like HAL may be able to overcome the pro-inflammatory phase better than passive exoskeletons to improve mobility by creating the neural circuits to bypass the level of injury hence inducing neuroplasticity.

For chronic SCI, HAL also showed greater improvements in both the 6MWT and 10MWT. HAL showed improvements of 75% in the 6MWT and Ekso only showed improvements of 34%. HAL was the only exoskeleton to show improvements in the 10MWT for chronic SCI patients with an increase of 92.4%. This suggests that the active exoskeleton HAL is able to regenerate lost neurons to induce neuroplasticity despite being more than a year since the injury.

All of the improvements in mobility were assessed in the short term. No long term follow-ups were carried out in the included studies. Khan et al looked at mid-term follow up using Rewalk which showed no significant changes 2-3 months post-rehabilitation. However, whether the initial pre/post exoskeleton rehabilitation improvements showed statistically significant changes were not mentioned. The lack of long term follow up serves as a limitation to this review.

## Secondary Outcomes

### Continence

Bladder and bowel continence are known complications that affect patients who have suffered an SCI. Neurogenic bowel affects nearly half of SCI patients and causes major disruptions to the patients’ social life and QoL.^46^ It is ranked as one of the highest priorities patients have after suffering an SCI.^34^ There are no effective treatments for patients to regain bladder function after an SCI.^47^ However, walking with an exoskeleton is thought to improve bowel and bladder function in patients after an SCI.^34^

In this review, Brinkemper et al was able to show significant improvements in 35 patients for both bladder and bowel function after locomotion with the exoskeleton HAL, an active exoskeleton, based on the Wexner score, a 20-point scoring system for continence. This suggests that rehabilitation with HAL was able to generate enough new neural circuits to bypass the level of the injury^4^ to induce neuroplasticity allowing the patients to actively be able to contract the external urethral sphincter and relax the urinary bladder to prevent incontinence.^47^ When the 35 patients were split into acute and chronic SCI, the Wexner score only showed significant improvements for the 22 chronic SCI patients and not the acute SCI patients.^34^ This suggests that HAL may be more effective in improving continence in patients with chronic SCI.

Van nes et al and Chun et al were able to demonstrate significant improvements using ReWalk. 21 patients using ReWalk, a passive exoskeleton, in van nes et al’s study improved based on the neurogenic bladder symptom score. This was in a group of patients with a median and range of 5.4 years (0.8 to 27 years) since the time of injury, again a more chronic group of SCI patients.^19^ Significant improvements were also observed in Chun et al’s study in a cohort of chronic SCI patients.^35^

Kim et al’s study using the H-MEX and Kerdraon et al’s study using the Atlante both did not show significant improvements in a cohort of chronic SCI patients.^33,41^ In addition, 2 patients in Williams et al’s study with Ekso also showed no significant improvements in a cohort of chronic SCI patients.^37^ This suggests that HAL and ReWalk may be better than H-MEX and Atlante at improving continence post SCI.

Both active and passive rehabilitation exoskeletons have shown positive evidence of improving continence post SCI but due to the vast variety of measurements used, it is very difficult to compare which exoskeletons have a greater impact on continence post SCI. However, the passive exoskeletons which don’t detect bioelectrical signals as a part of their rehabilitative mechanisms showed inconsistencies in improvements in continence. This suggests that their design may not be most efficient in neuro-regeneration therefore inducing less neuroplasticity with new neural circuits not able to bypass the level of injury.

Tamburella et al’s systematic review included Ekso and Indego as a part of their exoskeletons for urinary continence. They have also found that no significant changes were observed post exoskeleton rehabilitation.^44^ This coincides with the fact that passive exoskeletons may not be as consistent with improving urinary continence due to their mechanism of rehabilitation. In addition, 8 studies which all included passive exoskeletons in Tamburella et al’s systematic review looked at bowel functionality. Again, only one study showed significant improvements highlighting the inconsistencies and unreliability of the passive class of exoskeletons for bowel and bladder continence.^44^

However, as there was only one HAL study that looked at bowel and bladder continence, more studies with the active exoskeleton would be needed to draw concrete conclusions.

### Pain

It is estimated that 80% of patients with an SCI will suffer from pain syndromes. They can be nociceptive or neuropathic pain which both impact the patients’ QoL.^48^ 70% of patients will experience chronic pain and 5-37% of these patients will have pain that are refractory to treatment.^29^ Patients will often be prescribed a range of medications with common classes of drugs including anxiolytics, analgesic-narcotics and antidepressants which can have many side effects including constipation,^49^ which can further worsen the patient’s neurogenic bowel disease. In addition, social and psychological factors can contribute to the biological factors causing pain resulting a complex and multifactorial phenomenon.^50^

The 2 patients in Cruciger et al’s study with HAL, showed significant reductions in pain and also lead to the cessation of pain medications during the 4^th^ week of rehabilitation with HAL. There was an increase in pain intensity in the following week but then decreased shortly after. The 2 patients were both pain free and did not complain of pain at the one year follow-up nor needed to restart their pain medications despite “an excessive and long history of pain medication and concomitant treatment” before the rehabilitation.^29^

Exoskeleton rehabilitation activates a range of enzymes in the body to help regulate neuropathic pain. Promyelocyte kinase B signally pathway is stimulated resulting in an increase of glutamate decarboxylase in the spinal cord. This reduces pain by activating the gamma-aminobutyric acid (GABA) inhibition pathway and reduces the inflammation at the injury.^4^

The active exoskeleton HAL which can detect bioelectrical signals represented the majority of patients with significant reduction in pain. This could be due to the mechanism of the exoskeleton generating more neural circuits and neuroplasticity compared to passive exoskeletons which don’t detect bioelectrical signals.

However, a small sample size of only 3/134 patients who had pain showed significant reductions in pain post rehabilitation suggesting that both active and passive exoskeletons may not be the best rehabilitative tool for patients with an SCI.

### Quality of Life

SCI affects multiple body systems and can cause sudden changes in lifestyle for patients. Basic activities of daily living including dressing, washing, eating etc must be relearnt and can affect a patient’s QoL significantly. In addition, loss of bladder and bowel function and neuropathic pain can further decrease a patient’s QoL. SCI can also affect a patient’s mental health and suicide rates are thought to be 2 to 6 times greater than the general population.^51^

HAL, Rewalk and Ekso were the only exoskeletons to show significant improvements in QoL. Both HAL and Rewalk used the SF36 with the Ekso using the SF12. Both the SF12 and SF36 include physical and mental health domains. They are physical functioning, physical role limitation, bodily pain, vitality, social functioning, mental health, general health perception and emotional role limitation.^19^

HAL showed improvements in both the physical and mental health domain of the SF36. Rewalk showed improvements in 4/8 domains including bodily pain, social functioning, mental health and general health perception. Ekso showed significant improvements in the SF12 and as well as the physical aspects of the SF12. There is no mentioning of the mental aspect of the SF12 for the Ekso study.^16^ Tulsky et al looked at the SCI-QoL measurement system and has mentioned how the SF36 questionnaire can contain irrelevant questions that lack validity and how this does not gather meaningful information from SCI patients. Based on the SCI-QoL definition, QoL is separated into four main categories: Physical medical health, emotional health, social participation and physical functioning.^51^

Based on the four domains the SCI-QoL includes, the mechanism of the exoskeleton is unlikely to cause a difference in effect of the mental health and social participation aspects of QoL as unless there is also a concurrent improvement in physical function and physical medical health which improves the patients’ mental health. This is potentially shown in Maggio et al’s Rewalk study where improvements were seen in 4 out of 8 aspects of the SF36. One of these were physical factors (bodily pain) and this could have helped improve the mental aspects of QoL by improving social functioning, mental health and general health perception due to the reduction in pain.

Exoskeleton gait training can provide improvements in the emotional health of a patient due to a new outlook in their rehabilitation journey.^52^ It is unlikely that different types of exoskeletons will make a difference in the improvements in their emotional health and hence active and passive exoskeletons shouldn’t make a difference in the emotional health and social participation aspect of HrQoL unless there is a difference in improvements in physical medical health and physical functioning due mechanism of the exoskeleton.

However, Cruciger et al’s case study with 2 patients using HAL showed improvements in all domains of the SF36. This could suggest that the active exoskeleton actually improves the patients’ mental health based on its mechanism, but further studies will need to be carried out due to the indirect comparison and small sample size of the studies.

While improvements in mobility are often the primary focus of exoskeleton-assisted rehabilitation, it is equally important to consider the above secondary health outcomes that significantly impact the QoL in individuals with an SCI. This systematic review finds that neurogenic bladder and bowel dysfunction, which affect continence, are common after SCI and are associated with a high burden of care and social stigma.^34^ Regular upright positioning and movement through exoskeleton use may help improve bowel motility and bladder emptying, potentially reducing the need for invasive interventions. Similarly, neuropathic and musculoskeletal pain are prevalent and debilitating sequelae of SCI.^48^ Exoskeleton use may help alleviate pain by redistributing pressure, enhancing circulation, and promoting more natural postural alignment and movement patterns. Additionally, regular weight-bearing and dynamic activity have been associated with reduced spasticity, improved cardiovascular health, and better psychological well-being.^44^ By addressing these often-overlooked outcomes, exoskeleton-assisted rehabilitation may offer a more holistic therapeutic approach that extends beyond mobility to support comprehensive recovery and improved long-term health and independence.^44^

## Conclusion

In conclusion, HAL, the only active exoskeleton showed statistically significant improvements in mobility compared to all passive exoskeletons. HAL also showed consistent improvements in all secondary health outcomes including continence, pain and QoL suggesting that neuroplasticity was potentially induced across all aspects of the patient, showing the potential to address multiple complications of an SCI with just one rehabilitation regime. The passive exoskeleton Rewalk also has its strengths in improving secondary health outcomes but not mobility.

### Study Limitations

Our data only includes up to June 2023 due to logistical issues and delays in completing the manuscript. A large number of patients were included in the review over a range of different studies but there were no direct randomized control trials comparing an active and passive exoskeleton against each other. This is likely due to the logistical aspect of things where it is expensive for a rehabilitation centre to have 2 devices. The large majority of studies also scored poorly in the Newcastle Ottawa Scale and the Rob2 highlighting high levels of potential bias. Sample sizes for HAL was also much larger than other exoskeletons. In the future, we would like to see different rehabilitation centres working together to produce a randomised controlled trial comparing the active exoskeleton HAL to a passive exoskeleton. Only 3 studies included a follow-up for patients after rehabilitation with an exoskeleton^9,14,29^ with only Khan et al’s study showing no statistical difference between post training and the follow-up suggesting that the improvements in mobility were maintained. We would also like to see follow-ups post rehabilitation in future studies to see whether the effects of exoskeleton rehabilitation are maintained.

## Data Availability

Datasets are available upon reasonable request. *

## Appendix

**Table 1.** Inclusion and Exclusion Criteria for the Systematic Review.

Inclusion	Exclusion
Patients with an SCI that carried out rehabilitation with a lower limb exoskeleton	Neurological and neuro-degenerative pathologies e.g. stroke, multiple sclerosis, myelopathy
One or more of the outcome measures pre-and post-rehabilitation with a lower limb exoskeleton: 1. Mobility: 6 minute walking test (6MWT) and 10 metre walking test (10MWT) without the exoskeleton 2. Continence 3. Pain 4. Quality of life	Hybrid applications of exoskeleton rehabilitation e.g., functional electrical stimulations, epidural stimulation, transcutaneous spinal stimulation etc.

**Table 2.** An Overview of the Exoskeleton Devices Included in the Review.

Exoskeleton	Mechanism of Action	Classification
Hybrid assisted limb (HAL) ®	Bio-electrical signals are detected by emg-electrodes from the extensors and flexors of the hip and knee. The bio-electrical signals are amplified to allow synchronized voluntary drive of the patient to initiate limb movement.^29^	Active
Lokomat ®	A bilateral driven gait orthosis which is used together with a body weight support system. The exoskeleton will move the users legs in the sagittal plane with hip and knee actuators. Ankle dorsiflexion is supported by foot lifters during the swing phase. The hip and knee joint movements can be adjusted depending on the patients’ input which will depend on their neurological function.^53^	Passive
Ekso ®/Ekso GT ®	Users will shift their weight which will activate sensors in the device which will initiate steps.^8,16^	Passive
Hyundai medical exoskeleton (H-MEX)	A motherboard with hip and knee actuators is connected to a lithium battery. A passive ankle joint is also present. Bipedal gait is triggered by a button on the device. The leading foot is decided by comparison of the forces of both feet by optic force sensors.^41^	Passive
Rex Bionics	Without a biofeedback mechanism, a joystick controlled by the therapist controls the 10 actuators on the lower limb to move the user.^9^	Passive
ReWalk ®	Forward tilting of a sensor on the pelvic band results in the motors at the knees and hip to generate steps.^14^	Passive
Indego ®	A developed control system calculates the users centre of pressure (CoP) which is estimated using based on the user’s centre of mass relative to the horizontal plane and the distance the CoP and the forward ankle joint. Therefore, tilting the hip forwards and backwards results in movement in the relative directions.^17^	Passive
Atalante ®	The Atalante is an external, powered and motorized orthosis that is fully actuated with 12 actuated degrees of freedom. 3 at the hip, one at each knee and 2 at each ankle.^33^ Atalante also has a self-balancing system without the need of crutches. The exoskeleton can provide total assistance during the start of the training by facilitating symmetrical gait movements with decreasing facilitations as the patient improves.^54^	Passive
SuitX Phoenix ®	A tablet controls hip and knee movement. A handheld user interface allows the user to control actions like standing, walking and sitting.^38^	Passive
HANK	The exoskeleton has actuators including on the ankle used for joint flexion and extension. In addition, movement in other directions is restricted. There is also a battery pack, the main microprocessor and communication electronics in the backpack of the exoskeleton along with force sensing resistors to detect foot contact with the floor.^21^	Passive

**Table 3.** Results for all 6MWT.

Study	N =	Exoskeleton	Pre-Rehab	Post-Rehab	%Change	Statistical Significance
Grasmucke et al, 2017	55	HAL	97.81	146.34	49.6%	*P* ≤ 0.001
Chang et al, 2018	4	Ekso	50	67	34.0%	Significant – mean difference 16.9; 95% CI = (1.2, 32.5)
Gil-Agudo et al, 2023	11	HANK	114.67	183.56	60.1%	*P* = 0.00
Aach et al, 2023	50	HAL	135.58	233.33	72.1%	Significant (no *P* value)
Sczesny-Kaiser et al, 2015	11	HAL	86	149.73	74.1%	*P* < 0.001
Benson et al, 2015	3	ReWalk	62	68	9.7%	n/a
Zieriacks et al, 2021	88	HAL	115.5	185.2	60.3%	*P* ≤ 0.0001
Aach et al, 2014	8	HAL	70.1	163.3	133.0%	*P* = 0.05

**Table 4.** Mean Percentage Change for Exoskeletons That Produced Significant 6MWT Improvements.

Exoskeleton	Total Number of Patients	% Change in 6MWT
HAL	212	77.8%
HANK	11	60.1%
Ekso	4	34.0%

**Table 5.** Results for all 10MWT.

Study	N =	Exoskeleton	Pre-Rehab	Post-Rehab	%Change	Statistical Significance
Grasmucke et al, 2017	55	HAL	0.142	0.284	100.0%	*P* ≤ 0.001
Chang et al, 2018	4	Ekso	0.17	0.22	29.4%	Not significant – mean difference 0.04; 95% CI = (−0.02, 0.11)
Gil-Agudo et al, 2023	11	HANK	0.34	0.57	67.6%	*P* = 0.03
Aach et al, 2023	50	HAL	0.156	0.377	141.7%	*P* ≤ 0.000
Okawara et al, 2020	20	HAL	0.26	0.34	30.8%	*P* = 0.01
Benito-Penalva et al, 2011	39	Lokomat	0.063	0.25	296.8%	Not significant
Sczesny-Kaiser et al, 2015	11	HAL	0.25	0.5	100.0%	*P* = 0.001
Soma et al, 2021	1	HAL	0.12	0.45	275.0%	Significant (no *P* value)
Field-Forte and Roach 2011	14	Lokomat	0.17	0.18	5.9%	Not significant
Benson et al, 2015	3	ReWalk	0.0636	0.0882	38.7%	n/a
Zieriacks et al, 2021	111	HAL	0.143	0.274	91.6%	*P* ≤ 0.0001
Aach et al, 2014	8	HAL	0.28	0.5	78.6%	*P* ≤ 0.05
Cruciger et al, 2016	2	HAL	0.117	0.227	94.0%	n/a

**Table 6.** Mean Percentage Change Exoskeletons That Produced Significant 10MWT Improvements.

Exoskeleton	Total Number of Patients	% Change in 10MWT
HAL	255	90.5%
HANK	11	67.6%

**Table 7.** Results for Acute SCI 6MWT.

Study	N =	Exoskeleton	Pre-Rehab	Post-Rehab	%Change	Statistical Significance
Gil-Agudo et al, 2023	11	HANK	114.67	183.56	60.1%	*P* = 0.00
Aach et al, 2023	50	HAL	135.58	233.33	72.1%	Significant (no *P* value)
Zieriacks et al, 2021	47	HAL	127.1	245.1	92.8%	*P* ≤ 0.0001

**Table 8.** Mean Percentage Change Exoskeletons That Produced Significant Acute 6MWT Improvements.

Exoskeleton	Total Number of Patients	% Change in Acute 6MWT
HAL	97	82.5%
HANK	11	60.1%

**Table 9.** Results for Acute SCI 10MWT.

Study	N =	Exoskeleton	Pre-Rehab	Post-Rehab	%Change	Statistical Significance
Gil-Agudo et al, 2023	11	HANK	0.34	0.57	67.6%	*P* = 0.03
Aach et al, 2023	50	HAL	0.156	0.377	141.7%	*P* ≤ 0.000
Baunsguard et al, 2017	15	Ekso	0.283	0.35	23.7%	*P* = 0.041
Soma et al, 2021	1	HAL	0.12	0.45	275.0%	Significant (no *P* value)
Zieriacks et al, 2021	47	HAL	0.157	0.38	142.0%	*P* ≤ 0.0001

**Table 10.** Mean Percentage Change Exoskeletons That Produced Significant Acute 10MWT Improvements.

Exoskeleton	Total Number of Patients	% Change in Acute 10MWT
HAL	98	186.2%
HANK	11	67.6%
Ekso	15	23.7%

**Table 11.** Results for Chronic SCI 6MWT.

Study	N =	Exoskeleton	Pre-Rehab	Post-Rehab	%Change	Statistical Significance
Grasmucke et al, 2017	55	HAL	97.81	146.34	49.6%	*P* ≤ 0.001
Chang et al, 2018	4	Ekso	50	67	34.0%	Significant – mean difference 16.9; 95% CI = (1.2, 32.5)
Benson et al, 2015	3	ReWalk	62	68	9.7%	n/a
Zieriacks et al, 2021	74	HAL	111.5	158.8	42.4%	*P* ≤ 0.0001
Aach et al, 2014	8	HAL	70.1	163.3	133.0%	*P* < 0.05

**Table 12.** Mean Percentage Change Exoskeletons That Produced Significant Chronic 6MWT Improvements.

Exoskeleton	Total Number of Patients	% Change in Chronic 6MWT
HAL	137	75.0%
Ekso	4	34.0%

**Table 13.** Results for Chronic SCI 10MWT.

Study	N =	Exoskeleton	Pre-Rehab	Post-Rehab	%Change	Statistical Significance
Grasmucke et al, 2017	55	HAL	0.142	0.284	100.0%	*P* ≤ 0.001
Chang et al, 2018	4	Ekso	0.17	0.22	29.4%	Not significant – mean difference 0.04; 95% CI = (−0.02, 0.11)
Baunsguard et al, 2017	12	Ekso	0.296	0.366	23.6%	*P* = 0.322
Benson et al, 2015	3	ReWalk	0.0636	0.0882	38.7%	n/a
Aach et al, 2014	8	HAL	0.28	0.5	78.6%	*P* ≤ 0.05
Zieracks et al, 2021	74	HAL	0.146	0.29	98.6%	*P* ≤ 0.0001

**Table 14.** Mean Percentage Change Exoskeletons That Produced Significant Chronic 10MWT Improvements.

Exoskeleton	Total Number of Patients	% Change in Chronic 10MWT
HAL	137	92.4%

**Table 15.** Summary of Included Studies.

Study	Title	Design	Level of Evidence	Rehabilitation Program	Acute/Chronic SCI	ASIA Scores	Exoskeleton	Participants of Interest	Outcome Measures
Grasmucke et al, 2017	Against the odds: what to expect in rehabilitation of chronic spinal cord injury with a neurologically controlled hybrid assistive limb exoskeleton. A subgroup analysis of 55 patients according to age and lesion level	Prospective study	Level 3	5/week for 12 weeks	All chronic	18 ASIA A with ZPP, 24 ASIA C, 13 ASIA D	HAL	55	Mobility - 6MWT and 10MWT both without the exoskeleton
Maggio et al, 2022	Body representation in patients with Severe spinal cord injury: A pilot study on the promising role of powered exoskeleton for gait training	Randomised control trial	Level 2	5/week for 8 weeks	Unspecified	10 ASIA A, 11 ASIA B	Ekso GT	21	QoL – Short form 12
Chun et al, 2020	Changes in bowel function following Exoskeletal-assisted walking in persons with spinal cord injury: An observational pilot study	Prospective, observational study	Level 3	3-4/week for 12-14 weeks. At least 25 sessions (25-63 range)	All chronic	8 ASIA A, 2 ASIA B	ReWalk	10	Continence – Bristol Stool scale, SCI-QoL bowel management difficulties short form instrument
Kim et al, 2021	Effects of wearable powered exoskeletal training on functional mobility, physiological health and quality of life in non-ambulatory spinal cord injury patients	Prospective single group pilot study	Level 3	3/week for 10 weeks	All chronic	7 ASIA A, 1 ASIA B, 2 ASIA C	Hyundai medical exoskeleton (H-MEX)	10	QoL – Short form 36v2 Continence – Colon transit time
Sale et al, 2016	Effects on mobility training and de-adaptations in subjects with spinal cord injury due to a wearable robot: a Preliminary report	Pilot single case study	Level 4	3-4/week until 20 sessions	Unspecified	2 ASIA A 1 ASIA C	Ekso	3	Pain – Visual Analogue scale
Kerdraon et al, 2021	Evaluation of safety and performance of the self balancing walking system Atalante in patients with complete motor spinal cord injury	Prospective, observational study	Level 3	4/week for 3 weeks	All chronic	11 ASIA a	Atalante	11	Continence – Qualiveen score, neurogenic bowel dysfunction and Bristol Stool chart Pain – Neurogenic pain symptom inventory
Juszczak, Gallo and Bushnik 2018	Examining the effects of a powered exoskeleton on quality of life and secondary impairments in people living with spinal cord injury	Cross Sectional	Level 3	3-4/week for 8 weeks	Mixed/Unspecified	30 ASIA A, 5 ASIA B and 10 ASIA C	Indego	45	Continence – Self reports Pain – Self report using 1-10 scale QoL – Satisfaction with life scale
Williams et al, 2021	Exoskeleton gait training to improve lower urinary tract function in people with motor-complete spinal cord injury: A randomized pilot trial	Randomised pilot trial	Level 2	3/week for 12 weeks	1 acute 3 chronic Ekso both chronic 1 each for Lokomat	2 ASIA A, 2 ASIA B (1 A and 1 B for each)	Ekso and Lokomat	4	Continence – Qualiveen score
Chang et al, 2018	Exoskeleton-assisted gait training to improve gait in individuals with spinal cord injury: a Pilot randomized study	Parallel group RC pilot trial	Level 2	5/week for 3 weeks	All chronic	1 ASIA C, 3 ASIA D	Ekso	4	Mobility - 6MWT and 10MWT both without exoskeleton
Gil-Agudo et al, 2023	Exoskeleton‐based training improves walking independence in incomplete spinal cord injury patients: results from a randomized controlled trial	Prospective, randomised comparative study	Level 2	3/week for 5 weeks	All acute	8 ASIA C, 3 ASIA D	HANK	11	Mobility - 6MWT and 10MWT both without exoskeleton
Aach et al, 2023	Feasibility, safety, and functional outcomes using the neurological controlled hybrid assistive limb exoskeleton (HAL®) following acute incomplete and complete spinal cord injury – Results of 50 patients	Prospective study	Level 3	5/week for 12 weeks	All acute	3 ASIA A with ZPP, 30 ASIA C, 17 ASIA D	HAL	50	Mobility - 6MWT and 10MWT both without exoskeleton
Okawara et al, 2020	Gait ability required to achieve therapeutic effect in gait and balance function with the voluntary driven exoskeleton in patients with chronic spinal cord injury: a Clinical study	Non randomised open label single arm study	Level 3	2-5/week until 20 sessions	Mixed/Unspecified	2 ASIA a, 4 ASIA C, 6 ASIA D	HAL	12	Mobility – 10MWT without exoskeleton
Benito-Penalva et al, 2011	Gait training in human spinal cord injury using Electromechanical systems: Effect of device type and patient characteristics	Longitudinal study	Level 3	Daily for 8 weeks	32 acute 7 chronic	5 ASIA A and B, 18 ASIA C, 16 ASIA D	Lokomat	39	Mobility – 10MWT without exoskeleton
Sczesny-Kaiser et al, 2015	HAL® exoskeleton training improves walking parameters and normalizes cortical excitability in primary somatosensory cortex in spinal cord injury patients	Pilot prospective study	Level 3	5/week for 12 weeks	1 acute 10 chronic	5 ASIA A with ZPP, 1 ASIA B with ZPP, 5 ASIA C	HAL	11	Mobility - 6MWT and 10MWT both without exoskeleton
Soma et al, 2021	Hybrid assistive limb functional treatment for a patient with chronic incomplete cervical spinal cord injury	Case report	Level 4	5/week for 12 weeks	All acute	1 ASIA C	HAL	1	Mobility – 10MWT without exoskeleton
van nes et al, 2022	Improvement of quality of life after 2-month exoskeleton training in patients with chronic spinal cord injury	Prospective single group study	Level 3	24 sessions over 8 weeks	Mixed/Unspecified	20 ASIA A, 1 ASIA B	ReWalk	21	Continence – Neurogenic bladder symptom score and neurogenic bowel dysfunction score QoL – Short form 36 with walk wheel modification
Field-Forte and Roach 2011	Influence of a Locomotor training approach on walking speed and distance in people with chronic spinal cord injury: A randomized clinical trial	Randomised clinical trial	Level 2	5/week for 12 weeks	Unspecified	Unspecified	Lokomat	15	Mobility – 10MWT without exoskeleton
Sawada et al, 2021	Influence of body weight-supported treadmill training with voluntary-driven exoskeleton on the quality of life of persons with chronic spinal cord injury: a Pilot study	Pilot prospective study	Level 3	2-5/week until 20 sessions	Unspecified	2 ASIA A, 4 ASIA B, 8 ASIA C, 5 ASIA D	HAL	19	Pain – Neurogenic pain symptom inventory QoL – Short form 36 v2
Brinkemper et al, 2021	Influence of locomotion therapy with the wearable cyborg HAL on bladder and bowel function in acute and chronic SCI patients	Retrospective survey	Level 3	5/week for 12 weeks	13 acute, 22 chronic	5 ASIA A WITH ZPP, 1 ASIA B, 22 ASIA C, 7 ASIA D	HAL	35	Continence – Wexner Score, Cleveland Clinic constipation score
Benson et al, 2015	Lower-limb exoskeletons for individuals with chronic spinal cord injury: Findings from a feasibility study	Longitudinal prospective feasibility study	Level 4	2/week for 10 weeks	All chronic	3 ASIA A (1 with ZPP), 2 ASIA C	ReWalk	5 for non motor 3 completed without exoskeleton	Mobility – 6MWT and 10MWT both without exoskeleton Pain – Visual Analogue scale QoL – Assistive technology device predisposition assessment
Koljonen et al, 2021	Outcomes of a multicentre safety and Efficacy study of the SuitX phoenic powered exoskeleton for Ambulation by patients with spinal cord injury	Multicentre prospective cohort study	Level 3	20 Sessions	All chronic	24 ASIA A, 5 ASIA B, 11 ASIA C + D	SuitX Phoenix	40	Pain - unspecified
Postol et al, 2021	Physiotherapy using a free-standing robotic exoskeleton for patients with spinal cord injury: a Feasibility study	Feasibility pre-post interventional study	Level 4	2/week for 12 weeks	Unspecified	1 ASIA A 1 ASIA B	Rex Bionics	2	QoL – Short form 8
Zieriacks et al, 2021	Rehabilitation of acute vs chronic patients with spinal cord injury with a neurologically controlled hybrid assistive limb exoskeleton: Is there a difference in outcome?	Prospective study	Level 3	5/week for 12 weeks	47 acute 74 chronic	24 ASIA A with ZPP, 61 ASIA C, 36 ASIA D	HAL	121	Mobility – 6MWT and 10MWT both without exoskeleton
Aach et al, 2014	Voluntary driven exoskeleton as a new tool for rehabilitation in chronic spinal cord injury: a Pilot study	Pilot study	Level 3	5/week for 12 weeks	All chronic	4 ASIA A with ZPP, 1 ASIA B with ZPP, 2 ASIA C, 1 ASIA D	HAL	8	Mobility – 6MWT and 10MWT both without exoskeleton
Khan et al, 2019	Retraining walking over ground in a powered exoskeleton after spinal cord injury: a Prospective cohort study to examine functional gains and neuroplasticity	Prospective cohort study	Level 3	4/week for 12 weeks	All chronic	6 ASIA A, 2 ASIA B, 3 ASIA C, 1 ASIA D But 3 were lost to follow up and paper did not specify which patient it was	ReWalk	9	Pain – Numerical rating scale and McGill pain questionnaire
Baunsguard et al, 2017	Gait training after spinal cord injury: Safety, feasibility and gait function following 8 weeks of training with the exoskeletons from Ekso Bionics	Prospective study	Level 3	3/week for 8 weeks	15 acute 12 chronic	Non-specified for patients of interest	Ekso	27	Mobility – 10MWT without exoskeleton
Cruciger et al, 2016	Impact of locomotion training with a neurologic controlled hybrid assistive limb (HAL) exoskeleton on neuropathic pain and health related quality of life (HRQoL) in chronic SCI: a Case study	Case study	Level 4	5/week for 12 weeks	All chronic	2 ASIA A with ZPP	HAL	2	Mobility – 10MWT without exoskeleton Pain – Numerical rating scale QoL – Short form 36

**Table 16.** The Newcastle-Ottawa Scale Assessment for Non-randomised Studies.

Study	Selection	Selection of the Non-exposed Cohort	Ascertainment of Exposure	Demonstration that Outcome of Interest was Not Present at the Start	Comparability	Outcome	Was Follow-up Long Enough for Outcomes to Occur	Adequacy of Follow-up Cohorts	Total Score
	Representation of the Exposed Cohort				Comparability of Cohorts on the Basis of the Design or Analysis	Assessment of Outcome			
Grasmucke et al, 2017	*	-	*	*	-	*	-	-	4
Chun et al, 2020	*	-	*	*	-	-	-	-	3
Kim et al, 2021	*	-	*	*	-	-	-	-	3
Sale et al, 2016	*	-	*	*	-	*	-	-	4
Kerdraon et al, 2021	*	-	*	*	-	-	-	-	3
Juszczak, Gallo and Bushnik 2018	*	-	*	*	-	-	-	-	3
Aach et al, 2023	*	-	*	*	-	-	-	-	3
Okawara et al, 2020	*	-	*	*	-	-	-	-	3
Benito-Penalva et al, 2011	*	-	*	*	-	-	-	-	3
Sczesny-Kaiser et al, 2015	*	-	*	*	-	-	-	-	3
Soma et al, 2021	-	-	*	*	-	-	-	-	2
van nes et al, 2022	*	-	*	*	-	-	-	-	3
Sawada et al, 2021	*	-	*	*	-	-	-	-	3
Brinkemper et al, 2021	*	-	*	*	-	-	-	-	3
Benson et al, 2015	*	-	*	*	-	-	-	-	3
Koljonen et al, 2021	-	-	*	*	-	*	-	-	3
Postol et al, 2021	*	-	*	*	-	-	*	*	5
Zieriacks et al, 2021	*	-	*	*	-	*	-	-	4
Aach et al, 2014	*	-	*	*	-	*	-	-	4
Khan et al, 2019	-	-	*	*	-	-	*	*	4
Baunsguard et al, 2017	-	-	*	*	-	-	-	*	3
Cruciger et al, 2016	*	-	*	*	-	-	*	*	5

**Table 17.** Rob2 Assessment Domains for Randomized Studies.
